# Supplementary material for: Higher stability of novel live-attenuated oral poliovirus type 2 (nOPV2) despite the emergence of a neurovirulent double recombinant strain in Uganda
Source: Nat Microbiol. 2026 Jan 19;11(2):406–14. doi: 10.1038/s41564-025-02219-w (PMC12872467; doi:10.1038/s41564-025-02219-w)
Supplement: Supplementary file 1 — Reporting Summary [file 41564_2025_2219_MOESM1_ESM.pdf]

nature portfolio

Corresponding author(s): Javier Martin

Last updated by author(s): Oct 25, 2025

Reporting Summary

Nature Portfolio wishes to improve the reproducibility of the work that we publish. This form provides structure for consistency and transparency in reporting. For further information on Nature Portfolio policies, see our Editorial Policies and the Editorial Policy Checklist.

Statistics

For all statistical analyses, confirm that the following items are present in the figure legend, table legend, main text, or Methods section.

n/a	Confirmed	
☐	☒	The exact sample size (*n*) for each experimental group/condition, given as a discrete number and unit of measurement
☒	☐	A statement on whether measurements were taken from distinct samples or whether the same sample was measured repeatedly
☐	☒	The statistical test(s) used AND whether they are one- or two-sided<br>*Only common tests should be described solely by name; describe more complex techniques in the Methods section.*
☐	☒	A description of all covariates tested
☒	☐	A description of any assumptions or corrections, such as tests of normality and adjustment for multiple comparisons
☐	☒	A full description of the statistical parameters including central tendency (e.g. means) or other basic estimates (e.g. regression coefficient) AND variation (e.g. standard deviation) or associated estimates of uncertainty (e.g. confidence intervals)
☐	☒	For null hypothesis testing, the test statistic (e.g. *F*, *t*, *r*) with confidence intervals, effect sizes, degrees of freedom and *P* value noted<br>*Give P values as exact values whenever suitable.*
☒	☐	For Bayesian analysis, information on the choice of priors and Markov chain Monte Carlo settings
☒	☐	For hierarchical and complex designs, identification of the appropriate level for tests and full reporting of outcomes
☐	☒	Estimates of effect sizes (e.g. Cohen's *d*, Pearson's *r*), indicating how they were calculated

Our web collection on statistics for biologists contains articles on many of the points above.

Software and code

Policy information about availability of computer code

Data collection	Data for this study was produced by hardware and software associated with Illumina and Oxford Nanopore sequencing platforms. Metadata associated with study samples was provided in Microsoft Excel worksheets.
Data analysis	Simple linear regression statistical analysis and molecular clock-based inference of the nucleotide sequence data were done using GraphPad Prism (version 10.1.2) software. The dose required to cause paralysis in 50% of transgenic animals (PD50) was calculated by the Spearman–Karber method using Microsoft Excel software. Poliovirus sequencing data were processed and analysed using Geneious (version 10.2.6).

For manuscripts utilizing custom algorithms or software that are central to the research but not yet described in published literature, software must be made available to editors and reviewers. We strongly encourage code deposition in a community repository (e.g. GitHub). See the Nature Portfolio guidelines for submitting code & software for further information.

Data

Policy information about availability of data

All manuscripts must include a data availability statement. This statement should provide the following information, where applicable:

- Accession codes, unique identifiers, or web links for publicly available datasets
- A description of any restrictions on data availability
- For clinical datasets or third party data, please ensure that the statement adheres to our policy

All consensus poliovirus genomic sequences generated in this study have been deposited in the NCBI sequence database with GenBank accession numbers

PV576826-PV577058. Raw fastq NGS files generated in this study corresponding to double recombinant viruses and enterovirus sequences from sewage have been deposited in the NCBI Sequence Read Archive under project code Bio project number: PRJNA1295797. Metadata associated with each sequence (Lab ID, GenBank ID, sample type, collection date and location) are available as Supplementary Table 3. Source data are provided with this paper.

Research involving human participants, their data, or biological material

Policy information about studies with human participants or human data. See also policy information about sex, gender (identity/presentation), and sexual orientation and race, ethnicity and racism.

Reporting on sex and gender	N/A
Reporting on race, ethnicity, or other socially relevant groupings	N/A
Population characteristics	N/A
Recruitment	N/A
Ethics oversight	The study did not involve human participants but the analysis of viral nucleotide sequences obtained from viral isolates recovered from anonymous clinical (stools) and environmental samples (sewage). Clinical and environmental specimens analyzed in this study were collected as part of routine public health surveillance under the Global Polio Eradication Initiative (GPEI). Acute flaccid paralysis (AFP) collecting stool samples is a core component of Uganda's national poliovirus surveillance program, implemented by the Ministry of Health with technical support from the World Health Organization (WHO). These surveillance activities follow WHO-recommended protocols and are classified as non-research public health activities that do not require individual informed consent. Only de-identified viral sequencing data were analysed in this study. No personally identifiable information was collected or analysed.

Note that full information on the approval of the study protocol must also be provided in the manuscript.

Field-specific reporting

Please select the one below that is the best fit for your research. If you are not sure, read the appropriate sections before making your selection.

☒ Life sciences ☐ Behavioural & social sciences ☐ Ecological, evolutionary & environmental sciences

For a reference copy of the document with all sections, see nature.com/documents/nr-reporting-summary-flat.pdf

Life sciences study design

All studies must disclose on these points even when the disclosure is negative.

Sample size	No statistical methods were used to predetermine sample size. The study included all available clinical and environmental specimens collected through routine poliovirus surveillance activities conducted under the Global Polio Eradication Initiative (GPEI) in Uganda over the study period. Sample inclusion was determined by availability and detection of type 2 polioviruses, rather than experimental design, and is representative of field surveillance during and after nOPV2 campaigns.
Data exclusions	No data were excluded from analysis unless sequencing quality was insufficient for reliable interpretation (e.g., partial genome coverage or failed sequencing runs).
Replication	All virus isolation, molecular testing, and sequencing were performed using standard WHO-accredited protocols. Laboratory processes were carried out in reference laboratories that undergo annual WHO proficiency testing and accreditation. Key findings (e.g., presence of recombinant genomes) were independently verified through repeated sequencing or analysis pipelines where appropriate. No experimental replication was performed beyond standard confirmation procedures due to the descriptive nature of the surveillance data.
Randomization	Randomization was not applicable, as the study is based on observational surveillance data and not experimental assignment. Samples were tested in the order received, following standard laboratory workflows.
Blinding	Blinding was not applicable. Laboratory personnel conducting virus isolation and sequencing were aware of the sample origin (clinical or environmental), but no analytical decisions were influenced by these factors. Bioinformatic analysis was conducted based on sequence data without reference to the sample identity beyond metadata needed for interpretation.

Reporting for specific materials, systems and methods

We require information from authors about some types of materials, experimental systems and methods used in many studies. Here, indicate whether each material, system or method listed is relevant to your study. If you are not sure if a list item applies to your research, read the appropriate section before selecting a response.

Materials & experimental systems

n/a	Involved in the study
☒	☐ Antibodies
☐	☒ Eukaryotic cell lines
☒	☐ Palaeontology and archaeology
☐	☒ Animals and other organisms
☒	☐ Clinical data
☒	☐ Dual use research of concern
☒	☐ Plants

Methods

n/a	Involved in the study
☒	☐ ChIP-seq
☒	☐ Flow cytometry
☒	☐ MRI-based neuroimaging

Eukaryotic cell lines

Policy information about cell lines and Sex and Gender in Research

Cell line source(s)

Poliovirus isolation was performed using two WHO-recommended eukaryotic cell lines: L20B and RD. L20B cells, which are genetically engineered mouse L cells expressing the human poliovirus receptor CD155, provide a highly specific substrate for poliovirus replication and facilitate rapid differentiation from non-polio enteroviruses. RD cells (human rhabdomyosarcoma cell line), which support the growth of a broader range of enteroviruses, were used in parallel to ensure the sensitive detection of poliovirus. The cells were obtained from master cell banks stored at MHRA which are distributed through the World Health Organization's Global Polio Laboratory Network (GPLN) and are used routinely in accredited national and regional polio laboratories.

Authentication

Master cell banks are authenticated using validated molecular typing methods by WHO-designated reference laboratories prior to distribution. Receiving laboratories use these authenticated stocks and operate under WHO quality standards. Additional functional verification of cell lines is performed through annual WHO proficiency testing as part of the laboratory accreditation process.

Mycoplasma contamination

All laboratories involved in this study adhere to WHO and Good Laboratory Practice (GLP) standards. Regular quality control measures, including mycoplasma testing, are in place, and no contamination was identified during the study period.

Commonly misidentified lines
(See ICLAC register)

The RD and L20B cell lines used in this study are not listed in the ICLAC register of commonly misidentified cell lines. Both lines are widely validated and recognized within the Global Polio Eradication Initiative for their specificity and reliability in poliovirus isolation.

Animals and other research organisms

Policy information about studies involving animals; ARRIVE guidelines recommended for reporting animal research, and Sex and Gender in Research

Laboratory animals

Neurovirulence testing was performed using Tg66-CBA transgenic mice expressing the human poliovirus receptor (PVR), in accordance with WHO recommendations. Mice were 6–8 weeks old (50% male and 50% female). Tg66-CBA mice are the product of crossing Tg66 mice with CBA/J mice several times then selecting for homozygous CBA MHC genes and homozygous PVR.

Wild animals

No wild animals were involved in this study.

Reporting on sex

Both male and female Tg66 mice were included in approximately equal numbers to account for known sex-related differences in sensitivity to poliovirus neurovirulence. This balance is maintained to ensure robust and interpretable results and is consistent with WHO and ARRIVE recommendations.

Field-collected samples

Stool and sewage samples were collected through routine poliovirus surveillance activities under the Global Polio Eradication Initiative (GPEI) in Uganda. All samples were collected, transported, stored, and processed following WHO-recommended protocols and timelines to ensure optimal conditions for the reliable detection of live poliovirus. Environmental samples were obtained from established sewage surveillance sites, and clinical samples were collected through acute flaccid paralysis (AFP) surveillance systems coordinated by the Uganda Ministry of Health. No disturbance to wildlife, natural habitats, or ecosystems occurred during fieldwork.

Ethics oversight

Animal experiments were performed at the MHRA, under ethical approval from MHRA's Ethics and Human Materials Advisory Committees and the Animal Welfare and Ethical Review Body (AWERB). All procedures were conducted under UK Home Office Procedure Project Licence Number PPL PP6108158. The mice are housed in Individually Ventilated Cages (IVCs) or conventional cages, under controlled environmental conditions in accordance with the UK Animals (Scientific Procedures) Act 1986 and the Home Office Code of Practice for the Housing and Care of Animals Bred, Supplied or Used for Scientific Purposes (37). Ambient temperature is maintained between 20-24 degrees Celsius with relative humidity maintained between 45-65%. A daily 12:12 hour light/dark cycle with half an hour of half-light to mimic dawn and dusk, is provided to regulate circadian rhythms. Stocking density is carefully considered to ensure the mice are provided with sufficient floor space and to allow for provision of environmental enrichment in line with legislative standards. Environmental enrichment, including nesting material, refuges, wooden and disposable enrichment, is provided during weekly cage cleaning to encourage natural behaviours that are crucial for maintaining the health and wellbeing of the mice. Diet and water are provided ad libitum, and environmental parameters are continuously monitored to meet legislative and welfare requirements. Mice are routinely handled using refined handling techniques, including tunnel handling and cupping, to minimise stress and ensure high standards of animal welfare.

Note that full information on the approval of the study protocol must also be provided in the manuscript.

Plants

Seed stocks	*Report on the source of all seed stocks or other plant material used. If applicable, state the seed stock centre and catalogue number. If plant specimens were collected from the field, describe the collection location, date and sampling procedures.*
Novel plant genotypes	*Describe the methods by which all novel plant genotypes were produced. This includes those generated by transgenic approaches, gene editing, chemical/radiation-based mutagenesis and hybridization. For transgenic lines, describe the transformation method, the number of independent lines analyzed and the generation upon which experiments were performed. For gene-edited lines, describe the editor used, the endogenous sequence targeted for editing, the targeting guide RNA sequence (if applicable) and how the editor was applied.*
Authentication	*Describe any authentication procedures for each seed stock used or novel genotype generated. Describe any experiments used to assess the effect of a mutation and, where applicable, how potential secondary effects (e.g. second site T-DNA insertions, mosiacism, off-target gene editing) were examined.*
